# The role of renal denervation for the treatment of hypertension in patients with chronic kidney disease: a position paper of the Italian Society of Nephrology

**DOI:** 10.1007/s40620-025-02406-2

**Published:** 2025-09-08

**Authors:** Sandro Feriozzi, Yuri Battaglia, Calogero Lino Cirami, Concetta Gangemi, Gaetano La Manna, Loreto Gesualdo, Maria Lorenza Muiesan, Antonello Pani, Federico Pieruzzi, Flavio Ribichini, Stefano Taddei, Stefano Bianchi

**Affiliations:** 1 Nephrology, Fondazione Policlinico Universitario Campus Bio-Medico, Rome, Italy; 2 Department of Medicine, University of Verona, Verona, Italy; 3 Nephrology and Dialysis Unit, Pederzoli Hospital, Peschiera del Garda, Verona, Italy; 4 Nephrology, Dialysis and Transplantation Unit, Careggi University Hospital, Florence, Italy; 5 Division of Nephrology and Dialysis, University Hospital of Verona, Verona, Italy; 6 Nephrology, Dialysis, and Kidney Transplant Unit, IRCCS-Azienda Ospedaliera, Universitaria di Bologna, Bologna, Italy; 7 Department of Precision and Regenerative Medicine and Ionian Area, University of Bari “Aldo Moro”, 70124 Bari, Italy; 8 Centro studi diagnosi e cura dell’ipertensione arteriosa e del rischio cardiovascolare (IARC), Department of Clinical and Experimental Sciences, Division of Internal Medicine, University of Brescia, Brescia, Italy; 9 Nephrology, Dialysis and Transplantation Unit, ARNAS “G. Brotzu” Cagliari, CNR, Department of Medical Science and Public Health, University of Cagliari, Cagliari, Italy; 10 Nephrology, Fondazione IRCCS San Gerardo dei Tintori, Monza, Italy; 11 School of Medicine and Surgery, University of Milano-Bicocca, Milan, Italy; 12 Division of Cardiovascular Medicine, Department of Medicine, University of Verona, Verona, Italy; 13 Division of Internal Medicine, Department of Clinical and Experimental Medicine, University of Pisa, Pisa, Italy; 14 Nephrology and Dialysis Unit, ASL Nord Ovest Toscana, Livorno, Italy

**Keywords:** Uncontrolled and resistant hypertension, Renal denervation, Chronic kidney disease, Dialysis, Kidney transplant

## Abstract

Hypertension is a clinical condition associated with an increase in cardiovascular morbidity and mortality. In chronic kidney disease (CKD), hypertension is also a driver of faster disease progression. Correct and appropriate treatment with antihypertensive medication reduces the risk of cardiovascular events and slows kidney disease progression. However, not all hypertensive patients achieve the desired blood pressure goals despite multiple antihypertensive therapies, and these are defined as patients with uncontrolled or resistant hypertension. Renal denervation has been successfully proposed to mitigate the hyperactivity of the sympathetic nervous system in patients with uncontrolled/resistant hypertension. The guidelines of the European Society of Cardiology and the European Society of Hypertension include renal denervation as a therapeutic option for treating hypertension in patients with uncontrolled or resistant hypertension. However, in patients with CKD, diffusion of the procedure has been limited by concerns over possible adverse effects on kidney function. Over the last few years, the improvement of the device-based procedure and the growing experience accumulated in several Nephrology centers have increased the use of renal denervation in patients with CKD. In patients with uncontrolled/resistant hypertension with a significant reduction of glomerular filtration rate (< 40 ml/min/1.73 m^2^), on dialysis, or living with a kidney transplant, the procedure significantly reduces blood pressure values over time without negative effects on kidney function. Taking into account the effectiveness of renal denervation in patients with uncontrolled or resistant hypertension and CKD, and the lack of defined indications concerning renal denervation in CKD, the Italian Society of Nephrology has decided to publish a position paper reviewing the available evidence in the medical literature.

**Graphical abstract:**

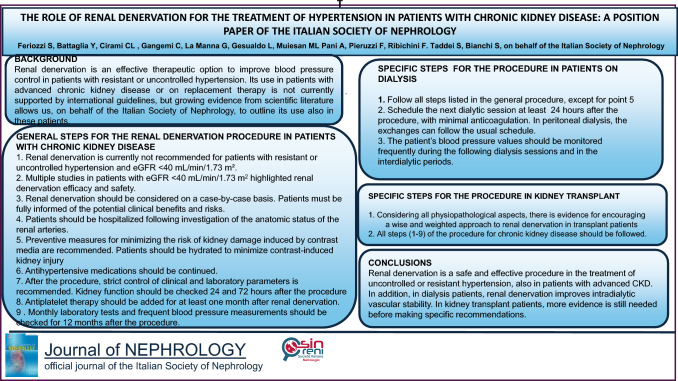

## Introduction

Hypertension (HTN) is the leading modifiable risk factor for global mortality, contributing significantly to cardiovascular, cerebrovascular, and renal complications [1]. Despite extensive treatment strategies, HTN remains a significant burden on healthcare systems and patients, particularly when it is resistant to standard therapy. Lowering blood pressure to recommended guideline values, achieved both by adhering to appropriate lifestyle measures and by pharmacological interventions, leads to a significant reduction of cardiovascular, cerebrovascular and renal events [2, 3]. The terms uncontrolled/resistant hypertension refer to elevated blood pressure values, despite the correct administration of three drugs, including a diuretic, at full dose. Uncontrolled or resistant hypertension affects 15–30% of patients with essential hypertension, and is associated with a significant increase in cardiovascular risk. Uncontrolled or resistant hypertension is more frequent in chronic kidney disease (CKD) and increases with the reduction of glomerular filtration rate (GFR). The presence of uncontrolled or resistant hypertension is also associated with faster progression of kidney damage [4].

Renal denervation is an interventional procedure aimed at ablating peripheral bundles of both afferent and efferent fibers of the sympathetic nervous system in the adventitia and perivascular tissue of renal arteries. This results in a long-term reduction of sympathetic activity and a significant reduction in blood pressure [5]. Renal denervation is an evolving interventional therapy for patients with uncontrolled or resistant hypertension. However, its use remains limited to select cases, as current guidelines do not support its widespread adoption.

In the last few years, many device-based procedures have been used to lower blood pressure in patients with uncontrolled or resistant hypertension, with the best evidence to date being achieved by a catheter-based technique. Data from sham-controlled trials investigating second-generation radiofrequency ablation catheters demonstrated long-lasting blood pressure-lowering efficacy in a broad range of patients, with and without concomitant blood pressure-lowering medications, including those with uncontrolled/resistant hypertension [6–9].

A crucial and challenging point would be having a marker, ahead of the procedure, for selecting patients who are likely to respond to renal denervation. Data from a systematic review of the literature suggest that pulse wave velocity correlates with the success of renal denervation. Indeed, stiffness parameters are strictly associated with post-procedural reduction of blood pressure. Therefore, assessing arterial stiffness before performing the procedure might help predict the response [10]. Based on the results of these studies, the 2024 Guidelines of the European Society of Cardiology (ESC) and European Society of Hypertension (ESH) for the management of elevated blood pressure and HTN included renal denervation as a therapeutic option for the treatment of HTN in patients with uncontrolled/resistant hypertension. These guidelines emphasize that renal denervation may be considered in patients with resistant hypertension who would prefer this procedure, and in those with uncontrolled hypertension on fewer than three drugs and with increased cardiovascular risk who would also prefer renal denervation, after a shared risk–benefit discussion and multidisciplinary assessment. Moreover, the same guidelines affirm that "Due to a lack of adequately powered outcomes trials demonstrating its safety and cardiovascular benefits, renal denervation is not recommended as a first-line blood pressure-lowering intervention for hypertension” Although there is no evidence to date suggesting that renal denervation may result in a deterioration of kidney function, treating hypertension in patients with moderate-to-severely impaired kidney function (eGFR < 40 ml/min/1.73 m^2^). [11, 12] with renal denervation is not recommended since these patients were excluded from sham-controlled trials.

Hypertension and the kidney are closely intertwined. HTN can cause and accelerate the progression of CKD. At the same time, CKD is a condition that promotes the onset and maintenance of the hypertensive state [13–16].

There is increasing interest among nephrologists in the possible role of renal denervation in treating HTN in patients with CKD and non-optimal control of blood pressure, as recommended by the current guidelines. While high-quality randomized controlled trials (RCTs) on renal denervation in hypertensive CKD patients are limited and do not consider the issue of chronic kidney disease, preliminary clinical studies suggest potential benefits in selected populations. Further research is necessary to confirm the efficacy and long-term safety of renal denervation in this group [17].

This position paper, endorsed by the Italian Society of Nephrology, aims to define the role of renal denervation in treating uncontrolled/resistant hypertension in patients with eGFR < 40 ml/min/1.73 m^2^, including those on dialysis and in kidney transplant patients. This document aligns with current national nephrology guidelines and consensus statements and seeks to provide a framework for decision-making in nephrology clinical practice.

## Rationale for renal denervation in patients with chronic kidney disease

Activation of the sympathetic nervous system increases blood pressure and induces several physiological reactions, which increase the heart rate and vasoconstriction, especially in the gastrointestinal tract and in the kidneys. The reduction of renal blood flow activates the Renin–Angiotensin–Aldosterone System (RAAS) and increases peripheral vascular resistance and sodium retention [18, 19].

Data from experimental studies and clinical investigations demonstrated that in patients with CKD, there is increased sympathetic nervous system activity, contributing to HTN and cardiovascular morbidity and mortality [12, 20]. CKD patients also have elevated levels of renin that, besides increasing blood pressure, may directly contribute to increased sympathetic nervous system activity [21]. Persistent renal ischemia can determine hyperactivity of the sympathetic nervous system due to the local release of adenosine and substance P [22] that may stimulate afferent nerves to the central nervous system. Clinical studies have shown that the serum levels of catecholamines are twice as high in patients with CKD compared to healthy subjects, while the availability of nitric oxide, a hypotensive substance, is significantly reduced [23]. In polycystic kidney disease, overactivity of the sympathetic nervous system is independent of kidney function [24]. Taken together, this demonstrates that sympathetic nervous system activation is both a cause and a consequence of GFR reduction. in turn, renal sympathetic nervous system activity impacts the central nervous system, triggering systemic effects, such as tachyarrhythmias, vasoconstriction, and insulin resistance.

Sympathetic nervous system activation is associated with HTN and is a relevant factor in accelerating the progression of kidney damage. Interestingly, catecholamines induce the proliferation of glomerular cells and interact with podocytes [25]. Activation of the sympathetic nervous system is also associated with an increase in inflammatory pathways that contribute autonomously to the progression of CKD [26].

Reducing sympathetic nervous system activity in patients with CKD is a mandatory clinical effort. Several antihypertensive medications can reduce sympathetic nervous system hyperactivity. Angiotensin-converting enzyme inhibitors (ACEi), Angiotensin II receptor blockers and beta blockers can minimize sympathetic nervous system hyperactivity in patients with CKD [27]. Moreover, in patients with heart failure, ACEi are effective in lowering hyperactivity of the sympathetic nervous system while also reducing the heightened plasma levels of circulating norepinephrine [28].

Conversely, in patients with CKD, calcium channel blockers exert no effect on the sympathetic nervous system [29]. Control of sympathetic nervous system status is associated with lower blood pressure values and improved cardiac and renal outcomes. A noteworthy aspect of renal denervation is its ability to reduce renin release. Indeed, renal denervation causes a decrease in plasma renin activity and aldosterone three months after the procedure, and higher baseline plasma renin activity values (> 0.65 ng/ml/h) are associated with a greater reduction of blood pressure [16].

An additional reason for recommending renal denervation in patients with CKD is provided by a study on renalase. Renalase is an enzyme that degrades circulating catecholamines, and it has been reported that its activity is reduced in CKD. CKD is characterized by an increase in sympathetic nervous system activity; therefore, reduced renalase levels and activity favor high levels of circulating catecholamines and result in adverse cardiovascular effects [30]. Reducing arterial blood pressure may slow the progression of kidney damage. A problem arises when multiple drugs are needed to control blood pressure. In a German study, a median of three medications was needed to control blood pressure in a population of patients with CKD [31]. Multiple drug therapy is a common practice in nephrology units; however, multi-drug therapy increases drug-related side effects and reduces patient adherence, leading to a negative impact on cardiovascular and renal outcomes [32].

Beyond its antihypertensive effects, renal denervation has shown promise in modifying key renal parameters. Studies report significant reductions in albuminuria and proteinuria, suggesting potential renoprotective effects. In patients with CKD and uncontrolled or resistant hypertension, renal denervation has been associated with stable or improved estimated GFR (eGFR) trajectories, contrary to initial concerns about kidney function deterioration. Notably, a meta-analysis of CKD patients undergoing renal denervation [33] demonstrated a clinically meaningful reduction in albuminuria (-25%) alongside preserved kidney function over 12–36 months of follow-up.

Renal denervation can be an appropriate choice for patients with CKD and uncontrolled/resistant hypertension, reducing sympathetic nervous system activity in one shot and for an extended period of time. A cross-sectional survey showed that among one thousand hypertensive patients, 30% preferred renal denervation to reduce their pill load [27, 33]. In Japan, a majority of younger male patients with extremely severe hypertension, poor adherence, and heart failure chose renal denervation rather than conventional medical therapy [34].

## Technical procedure of renal denervation in patients with chronic kidney disease

The renal denervation procedure poses no significant challenges to an expert interventionalist. Nevertheless, as with any other endovascular procedure, it requires method and training to enhance efficacy, minimize complications, and, in case of a complication, solve it in the best possible manner.

1. Patient preparation

Providing patients with adequate information is key, and they must be made aware of the need to continue medical therapy without changes unless indicated by the physician. Furthermore, the patient must be informed that the effects of the treatment will be apparent after 6 months, and may improve even after a year and for up to 3 years [35, 36]. Preventive hydration with saline 0.9% is recommended to reduce the risk of contrast-induced acute kidney injury regardless of the patient’s baseline kidney function. Intravenous hydration should be started a few hours prior to the renal denervation procedure, and the infusion rate should be set according to the left ventricular ejection fraction (LVEF); 1 ml/kg/h if the LVEF is normal, 0.5 ml/kg/h if LVEF < 40%.

The patient’s usual antihypertensive therapy must not be discontinued during the hospital stay or before the renal denervation procedure. As for any endovascular procedure, a pre-load with aspirin the day before (or a different antiplatelet in case of intolerance/contraindication) is advised. A single antiplatelet drug will be administered for 4 weeks (there is no scientific evidence supporting this recommendation).

Pre-procedural angiographic computed tomography is the ideal method to analyze renal artery anatomy, facilitating patient selection and exclusion, such as in cases of inadequate renal artery diameter or significant renal atherosclerotic disease and fibromuscular dysplasia. Moreover, pre-procedural computed tomography scans may detect anatomical variations, such as accessory renal arteries, and thus aid in procedural planning. Nevertheless, in case of limited computed tomography scan access, Doppler ultrasound can provide an idea of the renal artery flow in most cases, and the non-selective aortic angiogram (described below) at the start of the invasive procedure will provide all the necessary information.

Intravenous (IV) analgesia and sedation are mandatory before starting the renal denervation procedure because the technique, which is performed through either radiofrequency- or ultrasound-based energy, is painful and not tolerated unless the patient is adequately prepared [37].

2. The renal denervation procedure

Renal denervation is performed via the femoral vascular access through a percutaneous 6 Fr introducer in the case of radio-frequency-based catheters or 8 Fr when the ultrasound system is used. Unfractionated IV heparin is needed to maintain ACT > 250 s during the procedure.

In case of very high blood pressure values, continuous infusion of IV nitrates or sodium nitroprusside should be considered so as to perform the procedure within safe values of blood pressure to prevent vascular complications or hemodynamic decompensation such as pulmonary edema, angina or acute heart failure [37]. Patients with CKD are considered ideal candidates for renal denervation despite the lack of dedicated randomized studies. These patients require special attention to reduce the volume of the contrast medium. Patients on long-term dialysis may have tiny arteries, not suitable for the therapy, but this condition is very infrequent in our experience [38].

Dedicated guiding catheters for renal arteries are available on the market, and we advise their use rather than non-dedicated coronary catheters since their shorter length facilitates the procedure and reduces the amount of contrast media needed. The shape of the tip of the guiding catheter should be chosen according to the angulation of the emergence of the renal artery from the aorta. In most cases, a LIMA shape is the most suitable. Other catheters could be helpful when dealing with different anatomies, such as cases with upward artery take-offs. The Judkins right or multipurpose catheters may prove more convenient in these cases. Once selectively engaged, a complete angiogram of the kidney should be acquired to use as a roadmap for the procedure. Four to five ml of contrast at 5 ml/s is generally enough for a good angiogram. In most cases, fluoroscopy instead of fluorography can suffice to capture an acceptable quality of the images, thereby reducing radiation exposure. Importantly, gathering selective renal artery angiograms before initiating the renal denervation procedure remains essential for confirming the absence of abnormalities, such as fibromuscular dysplasia, that the antecedent CT scans may have missed [39].

Before starting the renal denervation procedure, establish a pre-defined strategy by selecting the target segments where the ablation catheter will be positioned. Such strategy differs substantially for radiofrequency- or ultrasound-based devices. The ablation target segments should include all the renal arterial branches with a diameter range between 3 and 8 mm outside the kidney parenchyma. Accessory renal arteries also carry sympathetic nerves and should be treated if the arterial diameter allows the insertion of the ablation catheter. Indeed, blood pressure reductions were significantly more noticeable when accessory arteries were also treated [37].

**Fig. 1:**
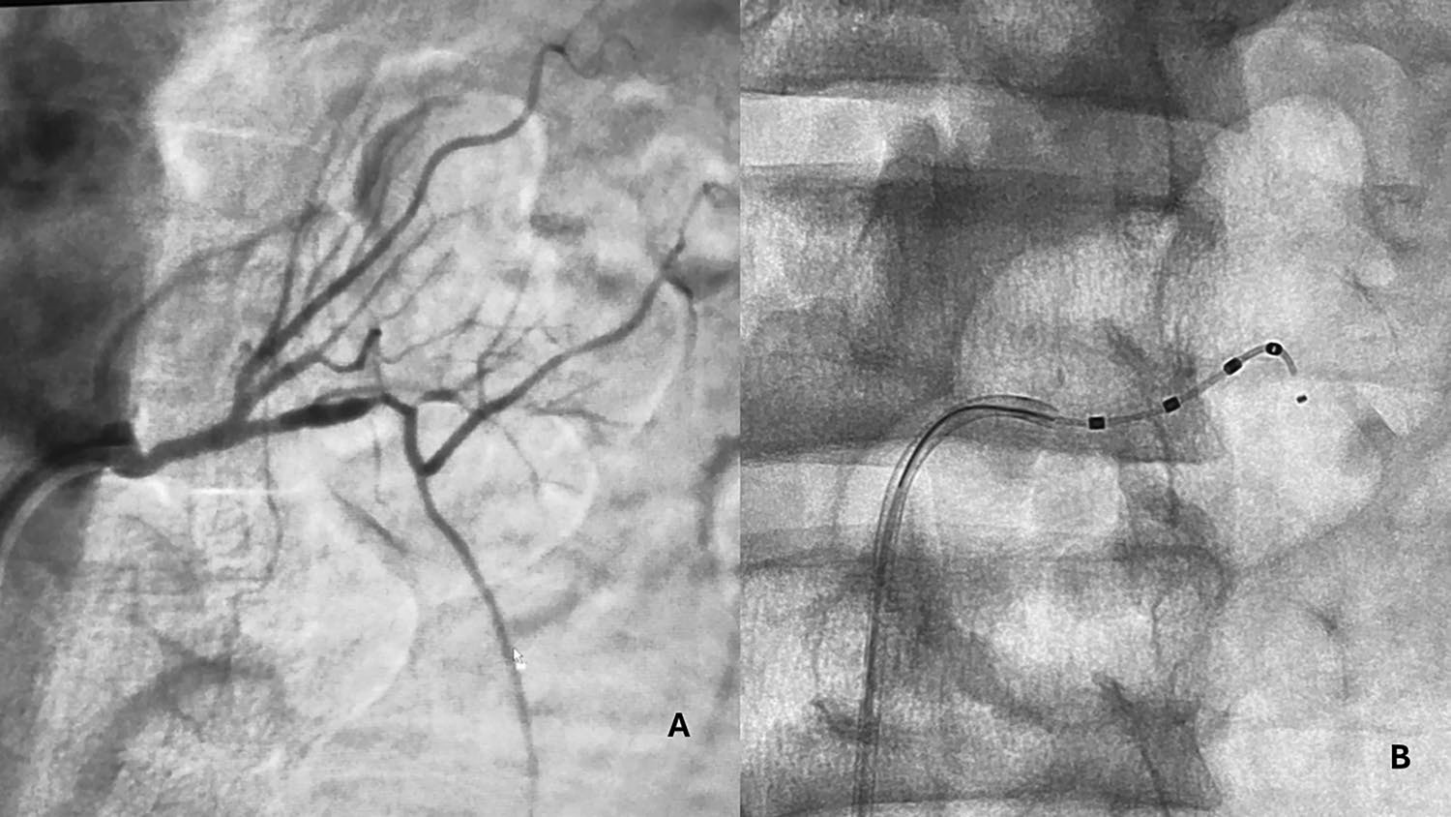
Renal denervation in a patient on chronic hemodialysis with severe hypertensive crisis. **A** Angiographic study of the renal artery. **B** The procedure of denervation with the spiral catheter. The lumens of the renal arteries were wide enough to allow for successful renal denervation. *Simplicity Spiral Catheter Denervation* (Courtesy of Fabrizio Chegai)

The Sypral renal denervation catheter from Medtronic © is a rapid exchange catheter that runs over a conventional 0.014″ coronary guidewire and navigates inside a 6 Fr guiding catheter. Targets of treatment for radiofrequency are the first portion of the arteries after their main bifurcation, the bifurcation site itself, and the vessel segment proximal to the bifurcation. The mid part of the main stem is also an adequate target (Fig. 1).

The angiographic pattern for radiofrequency treatment has been described as I–V–Y, which represents the main stem of the renal artery, the bifurcation, and the vessels beyond the first bifurcation [40]. Of note, the catheter never occludes the artery, and therefore, there is no interruption of blood flow to the kidney. Regardless of their position, size or tortuosity, all sites can be addressed with the same radiofrequency catheter. The Paradise US-ablation catheter from Recor Medical is a balloon that runs over a conventional 0.014″ coronary wire within an 8 Fr guiding catheter. The balloon size should equal the size of the vessel at the target treatment point to ensure adequate contact with the artery wall, causing a total occlusion of the vessel. The injection of contrast media must be used to confirm the absence of blood flow beyond the balloon.

Once the procedure is complete, regardless of the device used, repeat the selective angiography in the same baseline projection before disengaging the guiding catheter. Exclude vessel damage, and in case of evident vascular spasms, administer intra-arterial nitrates at the same dose used in coronary procedures. No further checks are needed at this stage.

## Renal denervation in patients with uncontrolled or resistant hypertension and advanced chronic kidney disease (eGFR < 40 ml/min/1.73 m^2^)

Nephrologists have long been interested in the availability of a procedure that can reduce both blood pressure values and sympathetic nervous system activity.

HTN is a driver of non-immunological progression of kidney disease and contributes to the high rate of cardiovascular complications of CKD. However, the goal of effective blood pressure control achieved with renal denervation has to be balanced with the feasibility and safety of the procedure. First, the anatomic status of the arterial renal vessels needs to be carefully assessed, and the potential for both acute and chronic worsening of kidney function induced by the administration of iodinated contrast media should be attentively considered.

Although current ESH and ESC guidelines [11, 12] do not recommend renal denervation as a first-line therapy, accumulating evidence in nephrology-specific populations suggests that its use in CKD patients with uncontrolled or resistant hypertension is both feasible and potentially beneficial. Recent studies have evaluated the safety of renal denervation in patients with advanced CKD, specifically analyzing changes in eGFR over time [41]. Their findings suggest renal denervation does not significantly accelerate kidney function decline; however, long-term data remain limited. Hering et al. [42] investigated patients with stage III or IV CKD, focusing on the variation of eGFR over time. In 15 patients, they did not observe a significant reduction of eGFR (31 ± 8.9 at baseline and 29.4 ± 7.3 ml/min/1.73 m^2^ after 6 months). Moreover, in a more restricted number of patients, the eGFR, evaluated with Cystatin C, showed a slight, though not significant increase, from 24.5 ± 10 to 31.5 ± 15. ml/min/1.73 m^2^ [36]. Mafhoud et al. [43] conducted a study in patients with resistant hypertension and micro- and macroalbuminuria, demonstrating that renal resistances were not modified and eGFR remained stable during the follow-up. It is noteworthy that albuminuria, a marker of worse cardiovascular and renal prognosis, was significantly reduced after renal denervation. In 2021, a meta-analysis of 11 single-center studies, involving 238 patients with CKD ranging from stage I to V, including those on HD, evaluated the effects of renal denervation on blood pressure, eGFR, and albuminuria. The results of the study confirmed that renal denervation effectively reduced blood pressure and albuminuria without any negative effects on eGFR [33]. A single-center Italian study published an interesting paper on this subject [38]. The authors investigated 40 patients with kidney disease and resistant hypertension and reported a significant reduction in blood pressure twelve months after renal denervation. Moreover, when the patients were divided into two groups with more or less than 45 ml/min/1.73 m^2^ of eGFR, the same effect on blood pressure control was observed in both groups. In patients with eGFR < 45 ml/min/1.73 m^2^, eGFR remained stable during the duration of the study. In a broad cohort of 475 patients with uncontrolled hypertension or resistant hypertension and CKD ( eGFR < 60 ml/min/1.73 m^2^) and 1505 without CKD (eGFR > 60 ml /min/1.73^2^), renal denervation significantly reduced 24-h ambulatory blood pressure in both groups. The data regarding the progression of kidney disease were interesting and encouraging. Indeed, during the first year, the group without CKD had a steeper decline in eGFR. After the first year, up until 3 years of follow-up, eGFR decline/year was similar in the two groups of patients. These results confirm the safety of renal denervation on kidney function, while the initial trend of the group without CKD could be due to the reduction of blood pressure values in more responsive kidneys. Moreover, the data on blood pressure control confirm the effectiveness of renal denervation in hypertensive patients independently of kidney function. There was no difference in the safety profile of the renal denervation procedure between groups [44].

Current scientific literature suggests that renal denervation is a promising therapy for uncontrolled/resistant hypertension in CKD, demonstrating consistent blood pressure reductions with a favorable safety profile. A meta-analysis [45] indicates no significant adverse renal effects in the short term, and preliminary evidence shows potential kidney function stabilization. However, studies including patients with advanced CKD remain limited, and none of them are RCTs, highlighting the need for further high-quality RCTs to establish definitive recommendations [46].

This position paper proposes that renal denervation should be considered in select CKD patients, particularly those who remain hypertensive despite medical therapy-optimized nephrology-based interventions (Table 1).

**Table 1:** Procedural steps for renal denervation in patients with advanced CKD (eGFR < 40 ml/min/1.73 m^2^)

1	One week before renal denervation, confirm office blood pressure and ABPM^a^ values
2	The patient must be hospitalized, a careful assessment of clinical and laboratory parameters must be carried out, and informed consent must be provided
3	Pre-procedural computed tomography angiogram has to be carried out
4	The dose of contrast medium should be the lowest necessary to perform the procedure effectively
5	Preventing or minimizing the risk of renal damage induced by the iodinated contrast media is recommended^b^
6	Antihypertensive medications should be taken as usual before the procedure, and effective antiplatelet therapy should be timely prescribed
7	Intravenous analgesia and sedation are mandatory before starting the procedure
8	Blood pressure, heart rate and clinical conditions should be checked accurately in the hours following the procedure
9	Serum creatinine should be checked 24 and 72 h after the procedure
10	The patient should maintain adequate hydration during hospitalization and after discharge unless contraindicated by clinical conditions
11	Monthly checks of laboratory tests and frequent blood pressure measurements for 3–6–12 months (office blood pressure) after the procedure should be scheduled. Aspirin use is recommended at least one month after renal denervation in order to avoid thrombotic events

Recommendations from a panel of experts

^a^Ambulatory Blood Pressure Monitoring

^b^Measures to prevent radio-contrast injury (Guidelines “Italian Society Nephrology”, “Italian Society Medical and Intervention Radiology and Italian Association of Medical Oncology” chrome-extension://efaidnbmnnnibpcajpcglclefindmkaj/https://sirm.org/wp-content/uploads/2021/04/316-Documento-intersocietario-SIRM-SIN-AIOM-2020-prevenzione-danno-renale-da-mdc.pdf

## Renal denervation in patients with uncontrolled or resistant hypertension on dialysis

In dialysis patients, hypertension remains one of the most challenging complications, often exacerbated by volume overload, vascular calcification, and increased sympathetic nervous system activity. Despite strict sodium and fluid management, blood pressure control usually requires polypharmacy, which in turn increases the risk of intradialytic hypotension. Emerging data suggest that renal denervation may offer an alternative approach by reducing sympathetic nervous system overactivity, thereby improving blood pressure stability without compromising hemodynamic tolerance during dialysis [47].

On the other hand, dialysis patients’ compliance with the therapy is compromised by their clinical conditions, the burden of the daily number of pills, and the difficulty in controlling the interdialytic increase of body weight. Therefore, nephrologists have started performing renal denervation in these patients when other therapeutic options did not allow them to achieve satisfactory blood pressure control. The anatomic conditions of renal arteries in patients on dialysis (stenosis of the renal arteries, small kidneys) have not precluded the procedure in safe conditions (Fig. 1). To date, there is a small but increasing number of published papers on renal denervation in dialysis patients.

Schlaich et al. [48] proposed the renal denervation procedure to 12 patients on hemodialysis with uncontrolled/resistant hypertension. In 3 patients, the procedure was impossible due to atrophy of the renal arteries. In the 9 treated patients, the office systolic blood pressure was significantly reduced by renal denervation from a mean of 166 ± 16 to 138 ± 17 mmHg at 12 months. The test for noradrenaline spillover was available in 5 patients, showing a significant reduction at 12 months; the same trend was recorded for muscle sympathetic activity.

Ott et al. [49] performed renal denervation in 6 patients on hemodialysis and observed a significant reduction of blood pressure in all the treated patients. In addition, the dry body weight of these patients decreased by about 1.8 kg after the procedure. This result, always challenging to obtain in real life, is possibly explained by better cardiovascular conditions allowing a more efficient dialysis treatment. If confirmed by future studies, renal denervation could be a strong recommendation in uncontrolled or resistant hypertension patients on hemodialysis who do not achieve satisfactory interdialytic dry weight control. A study performed in Italy involving 24 patients on hemodialysis treated with renal denervation reported a significant reduction in systolic and diastolic blood pressure in a six-month follow-up. Blood pressure values were reduced both in the day and night profiles, and no periprocedural complications occurred [50].

Mazza et al. [51] published a successful case of renal denervation in a patient on hemodialysis with resistant hypertension, and provided a summary of the available literature. Despite the limited number of studies, the positive trend in the effectiveness and safety of renal denervation was confirmed.

Recently, Gangemi et al. [52] published an interesting experience on 14 dialysis patients, 7 of whom were on peritoneal dialysis. Renal denervation was effective in reducing blood pressure and the number of antihypertensive medications, also in peritoneal dialysis patients.

When evaluating the results of renal denervation in patients on hemodialysis, we should also consider that renal denervation allows us to reduce the number of antihypertensive drugs. It is well known that antihypertensive medications are associated with hypotensive episodes [53]. Therefore, renal denervation might indirectly reduce the number of hypotensive episodes, improving dialysis efficiency (Table 2).

**Table 2:** Procedural steps for renal denervation in dialysis patients

1	One week before renal denervation, confirm office blood pressure and ABPM^a^ values
2	*Which patients are eligible* (a) Those taking three or more drugs but whose blood pressure is above the recommended target (b) Those for whom antihypertensive therapy controls blood pressure but is associated with frequent intra-dialytic hypotension
3	*What to do before the procedure* (a) The patient agrees to undergo renal denervation and provides signed informed consent (b) The anatomic status of the renal vessels must be accurately investigated to assess the feasibility of the procedure (c) Antihypertensive medications should be taken as usual before the procedure, and effective antiplatelet therapy should be timely prescribed
4	*What to do after the procedure* (a) The patient must be hospitalized (b) Strict control of clinical parameters must be carried out (blood pressure, heart rate) (c) The next dialytic session should be scheduled at least 24 h after the procedure, with minimal anticoagulation. In peritoneal dialysis, the exchanges can follow the usual schedule (d) Follow-up has to be established with strict blood pressure evaluations (3–6–12 months) scheduled during the dialysis and in the interdialytic period (e) Aspirin use is recommended for at least one month after renal denervation in order to avoid thrombotic events

Recommendations from a panel of experts

^a^Ambulatory blood pressure monitoring

## Renal denervation in patients with kidney transplant and uncontrolled or resistant hypertension

Post-transplant hypertension remains highly prevalent, affecting 70–90% of kidney recipients, and is linked to calcineurin inhibitors, RAAS activation, and residual sympathetic nervous system hyperactivity from native kidneys. Hypertensive kidney transplant patients often do not respond effectively to antihypertensive drugs due to persistent sympathetic nervous system dysregulation [54]. Preliminary studies [55] indicate that renal denervation performed on native renal arteries can effectively lower nocturnal blood pressure and restore a normal circadian rhythm, with potential benefits for long-term graft function.

In a recently published study, 9 hypertensive kidney transplant patients underwent renal denervation and were compared with 9 hypertensive kidney transplant patients receiving only medical therapy. After six months, the group treated with renal denervation showed a significant reduction in office systolic and monitored nocturnal blood pressure, while no change was observed among patients receiving medical therapy alone. In the renal denervation group, more patients converted from non-dippers to dippers. There were no adverse safety events in either group.

It is noteworthy that the authors report that the procedure was feasible in native kidney vessels, although the mean caliber of renal arteries was reduced [55]. Renal denervation in kidney transplant patients has been successfully reported in other anecdotal reports [56, 57].

Hypertension may be an unfavorable factor in transplant patients thus contributing to high cardiovascular morbidity and mortality. Therefore, renal denervation may be considered a therapeutic option to preserve the function of the transplanted kidney as long as possible in patients with uncontrolled/resistant hypertension (Table 3).

**Table 3:** Procedural steps for renal denervation in patients with renal transplant

1	One week before renal denervation, confirm office blood pressure and ABPM^a^ values
2	*Which patients are eligible* (a) Those who adhere to antihypertensive drug treatment (b) Those receiving the lowest required dose of steroids and calcineurin inhibitors
3	*What to do before the procedure* (a) The patient agrees to undergo renal denervation and provides signed informed consent (b) The patient’s renal function and anatomic status of the native renal arteries must be accurately investigated. Graft renal artery stenosis must be excluded (c) Antihypertensive medications should be taken as usual before the procedure, and effective antiplatelet therapy should be timely prescribed d) Prevent or minimize the risk of kidney damage induced by the iodinated contrast media^b^
4	*What to do after the procedure:* (a) The patient must be hospitalized (b) Strict evaluation of clinical parameters (blood pressure, heart rate, diuresis) (c) Serum creatinine check is mandatory at discharge and after 72 h (d) Frequent blood pressure measurements, monthly check up of renal function and an accurate review of the antihypertensive therapy (blood pressure office and ABPM§^a^) for at least 3–6–12 months after the procedure (e) Aspirin use is recommended for at least one month after renal denervation in order to avoid thrombotic events

Recommendations from a panel of experts

^a^Ambulatory blood pressure monitoring

^b^Measures to prevent radio-contrast injury (Guidelines “Italian Society Nephrology”, “Italian Society Medical and Intervention Radiology and Italian Association of Medical Oncology” chrome-extension://efaidnbmnnnibpcajpcglclefindmkaj/https://sirm.org/wp-content/uploads/2021/04/316-Documento-intersocietario-SIRM-SIN-AIOM-2020-prevenzione-danno-renale-da-mdc.pdf

However, the limited available experience regarding renal denervation in transplant patients should be augmented, with more extensive studies comparing renal denervation with a sham control group.

## Conclusions

Renal denervation is a safe and effective procedure for the treatment of uncontrolled or resistant hypertension. It can also be performed in advanced CKD (eGFR < 40 ml/min/1.73 m^2^), reducing blood pressure and slowing the progression of kidney damage. In dialysis patients, renal denervation improves intradialytic vascular stability. In kidney transplant, limited but promising data in the literature suggest considering renal denervation in uncontrolled or resistant hypertension (Table 4). This cautious but proactive approach for the indications of renal denervation in patients with CKD is currently shared by the nephrology community [58] and it is shown in a list of graded recommendations (Table 5).

**Table 4:** Position points on the role of renal denervation for hypertension in CKD in patients undergoing dialysis or renal transplant

(A) Patients with eGFR > 40 ml/min/1.73 m^2^ not on renal replacement therapy
1. Second-generation catheter-based renal denervation using radiofrequency or ultrasound may be considered for patients with uncontrolled or resistant hypertension and eGFR > 40 ml/min/1.73 m^2^, provided they have undergone a thorough risk–benefit assessment and multidisciplinary consultation. However, current guidelines do not recommend renal denervation as a first-line therapy for this population [11, 12]
2. Long-term follow-up data (up to 3 years) from randomized controlled trials of renal denervation performed in patients with resistant or uncontrolled hypertension and eGFR > 40 ml/min/1.73 m^2^ have not reported worsening of kidney function beyond the expected rates in patients with mild to moderately reduced kidney function. Moreover, there are no reported procedure-related serious adverse events beyond the usual risk for femoral arterial access procedures
3. Renal denervation should be performed in centers with adequate experience and a medium-to-high volume of endovascular interventional activity
(B) Patients with eGFR < 40 ml/min/1.73 m^2^ not on renal replacement therapy
1. Due to the lack of adequately powered trials, renal denervation is currently not recommended for patients with resistant or uncontrolled hypertension and eGFR < 40 ml/min/1.73 m^2^, according to the Guidelines of International Scientific Societies
2. Nonetheless, in the last few years, numerous studies carried out in patients with resistant or uncontrolled hypertension and eGFR < 40 ml/min/1.73 m^2^ have highlighted how the efficacy of renal denervation, in terms of blood pressure reduction and safety profile, is similar to what is observed in patients with more preserved or normal kidney function
3. For patients with uncontrolled/resistant hypertension and eGFR < 40 ml/min/1.73 m^2^, renal denervation should only be considered on a case-by-case basis. Patients must be fully informed about the potential benefits, current evidence limitations, and possible risks
4. Patients must be hospitalized, and their kidney function and anatomic status of the renal arteries must be accurately investigated
5. Antihypertensive medications should be taken as usual before the procedure, and effective antiplatelet therapy should be timely prescribed
6. Patients should be hydrated to prevent or minimize the risk of contrast media renal injury
7. After the procedure, strict evaluation of clinical and laboratory parameters is recommended
8. Kidney function assessment is mandatory: serum creatinine at 24 and 72 h after the procedure must be measured
9. Aspirin use is recommended for at least one month after renal denervation to prevent possible thrombosis of the renal arteries
10. Monthly evaluation of laboratory tests, and frequent blood pressure measurements at 3–6–12 months after the procedure should be scheduled. An accurate check of the antihypertensive therapy is necessary over time
(C) Patients on dialysis
1. Renal denervation could be taken into consideration as a therapeutic option in patients with uncontrolled or resistant hypertension. It may also be considered when antihypertensive therapy allows adequate blood pressure control but the patient manifests frequent intra-dialytic cardiovascular instability with frequent hypotensive episodes and does not reach a satisfactory dry weight despite correct management of the dialysis sessions
2. Patients must be hospitalized
3. Antihypertensive medications should be taken as usual before the procedure, and effective antiplatelet therapy should be timely prescribed
4. The next dialytic session should be scheduled at least 24 h after the procedure, with minimal anticoagulation. In peritoneal dialysis, the exchanges can follow the usual schedule
5. The anatomic status of the renal arteries must be accurately investigated
6. The patients’ blood pressure values should be monitored frequently during the following dialysis sessions and in the interdialytic periods
7. Aspirin use is recommended for at least one month after renal denervation to prevent possible thrombosis of the renal arteries
(D) Transplant patients
The renal denervation procedure has a consistent physiopathologicial basis in patients with uncontrolled or resistant hypertension. However, the literature evidence is anecdotal and does not allow for definitive recommendations to be made
Taking all these aspects into consideration, we believe there is evidence for encouraging a wise and weighted approach to renal denervation in transplant patients

**Table 5:** Recommendations for renal denervation in chronic kidney disease

Patient categories	Scale of recommendation	Comments
Patients with eGFR > 40 ml/min/m^2^	✪✪✪	Safe and effective procedure
Patients with eGFR < 40 ml/min/m^2^	✪✪	Safe and effective after a thorough clinical assessment of the patient
Patients on dialysis	✪✪	Safe and effective after a thorough clinical assessment of the patient and the dialytic schedules
Patients with a kidney transplant	✪	Extremely limited evidence

✪ semiquantitative indication marker

Based on the reported data, we suggest that a prospective registry on renal denervation for chronic kidney disease could be very interesting and scientifically valuable for establishing the indications and role of renal denervation in patients with chronic kidney disease. This would make it possible to collect data on the effectiveness of the short- and long-term results of the procedure on renal and cardiovascular outcomes.

**Advisory Board**

*Panel*: Chegai Fabrizio Viterbo, Calanna Massimo Messina, Casamassima Emanuele Firenze, Cutruzzulà Roberta Firenze, De Nicola Luca Napoli, Ferrantelli Angelo Palermo, Ferraro Pietro Manuel Verona, Fiorini Fulvio Rovigo, Roccatello Dario Torino, Gallo Paolo Roma, Magnoni Giacomo Bologna, Navajas Francisca Roma, Nazzaro Livia Roma, Paletta Domenico Viterbio, Panuccio Vincenzo Reggio Calabria, Pisani Antonio Napoli, Polci Rosaria Ascoli Piceno, Pepa Matteo Ascoli Piceno, Pucci Giacomo Terni, Ranghino Andrea Ancona, Santoro Domenico Messina, Savio Daniele Torino, Tavella Domenico Verona, Ussia Gianpaolo Roma, Valente Mauro Ancona, Vecchi Luigi Terni, Vettoretti Simone Monza.

## Data Availability

All data generated or analyzed during this study are included in this published article.
